# Brain Connectivity Signature Extractions from TMS Invoked EEGs

**DOI:** 10.3390/s23084078

**Published:** 2023-04-18

**Authors:** Deepa Gupta, Xiaoming Du, Ann Summerfelt, L. Elliot Hong, Fow-Sen Choa

**Affiliations:** 1 Computer Science and Electrical Engineering, University of Maryland Baltimore County, 1000 Hilltop Circle, Baltimore, MD 21227, USA; 2 Maryland Psychiatric Research Center, University of Maryland School of Medicine, 655 W. Baltimore Street, Baltimore, MD 21201, USA

**Keywords:** brain, network, connectivity signatures, EEG, electroencephalography, signal processing, machine learning, schizophrenia, neuro-psychiatric diagnosis

## Abstract

(1) Background: The correlations between brain connectivity abnormality and psychiatric disorders have been continuously investigated and progressively recognized. Brain connectivity signatures are becoming exceedingly useful for identifying patients, monitoring mental health disorders, and treatment. By using electroencephalography (EEG)-based cortical source localization along with energy landscape analysis techniques, we can statistically analyze transcranial magnetic stimulation (TMS)-invoked EEG signals, for obtaining connectivity among different brain regions at a high spatiotemporal resolution. (2) Methods: In this study, we analyze EEG-based source localized alpha wave activity in response to TMS administered to three locations, namely, the left motor cortex (49 subjects), left prefrontal cortex (27 subjects), and the posterior cerebellum, or vermis (27 subjects) by using energy landscape analysis techniques to uncover connectivity signatures. We then perform two sample *t*-tests and use the (5 × 10^−5^) Bonferroni corrected *p*-valued cases for reporting six reliably stable signatures. (3) Results: Vermis stimulation invoked the highest number of connectivity signatures and the left motor cortex stimulation invoked a sensorimotor network state. In total, six out of 29 reliable, stable connectivity signatures are found and discussed. (4) Conclusions: We extend previous findings to localized cortical connectivity signatures for medical applications that serve as a baseline for future dense electrode studies.

## 1. Introduction

It is estimated that mental health may cost the global economy USD 16 trillion until 2030, which is more than cancer, diabetes, and respiratory diseases combined. Twelve billion working days per year are lost as sick leave due to mental health illnesses [1]. There have been continuous investigations about the relationships between psychiatric disorders and brain connectivity abnormality and their correlation has been increasingly accepted by the community [2,3,4,5]. Extracting brain connectivity signatures can be useful for identifying patients and separating the patient and control groups. They can also be used for monitoring disorders and their treatment efficacies. 

For therapeutic treatment of mental health ailments, as an alternative to drugs, TMS is an established FDA treatment for mental health illnesses such as major depression disorder, migraines, obsessive-compulsive disorder, and smoking cessation. Since it is noninvasive and rarely has adverse effects, TMS has also been used as a diagnostic tool to measure connections between different regions [6]. Working together with other imaging tools, TMS applications have been further extended to areas that cover brain connectivity and cognitive, perceptual, behavioral, and therapeutic investigations [7,8,9,10,11,12,13]. Hence, a neuroimaging modality like electroencephalography (EEG) that gathers brain activity data from the scalp at a high temporal resolution [14] becomes a suitable tool for the study of the causal events of TMS-induced brain activity [15,16]. The fast connectivity signatures (within less than a second or a few seconds) may offer new paths to evaluate neuropsychiatric disorders. TMS combined with EEG techniques can unravel critical network signatures relevant to schizophrenia [17,18]. 

In this work, we process EEG, gathered from a clinical population of schizophrenia patients and healthy controls, by using cortical source localization-based energy landscape analysis, in response to a TMS subthreshold pulse administered to three different cortical sites: a. the left motor cortex (49 subjects), b. left prefrontal cortex (27 subjects), and c. the posterior cerebellum (vermis) (27 subjects). As per previous literature, it has been reported that the EEG alpha power characterizes abnormalities in schizophrenia [19,20]. Furthermore, our measurements were done when subjects were in a resting state, involving no tasks, with closed eyes; the alpha brain wave is the dominated brain wave in such conditions. Hence, we focus on alpha-wave-based localized EEG in response to three TMS subthreshold stimulation sites, to extract brain connectivity–network energy states that can significantly distinguish schizophrenia and healthy individuals. Cerebellum, prefrontal, and motor cortical stimulations have been previously studied in schizophrenia resting state functional connectivity for clinical efficacy [21,22,23]. In this study, we expand it further by applying an energy landscape [24,25,26,27,28] to source-localized EEG signals in response to TMS to these three sites. The obtained connectivity signatures are among derived brain regions of interest (ROIs), which can provide neurophysiology implications as described in the Methods section. Energy landscape analysis has been used with EEG at the scalp in the past [29], although the obtained signature connectivity is among different electrodes without source localization to brain regions. With this methodology, we were able to extend our previously reported insight further where EEG response to the TMS that was administered to multiple stimulation locations and ERP N100, a component of which was characterized as a significant signature [21]. Here, we observe that the posterior cerebellar stimulation was the strongest site for invoking stable connectivity networks and that the left motor cortex stimulation invoked a sensorimotor network state. These stable networks significantly distinguished patients from controls, which is further discussed in the results and discussion section. 

## 2. Materials and Methods

**Figure 1:**
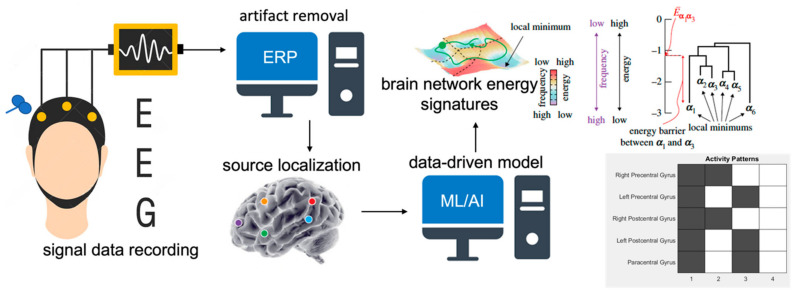
EEG data recording in response to TMS are preprocessed for artifact removal and ERP extraction. Scalp ERP is processed for cortical source localization. Then, machine learning analysis of localized cortical source current density values is done to get brain region network energy values. Statistical analysis of the obtained brain network energies is performed for the patients and healthy control groups to select the relevant extracted signature for plausible clinical application.

Non-invasive EEG signal data were gathered from the scalp in response to TMS administered over three different cortical sites, namely, the left motor cortex, left prefrontal cortex, and the posterior cerebellar region. The continuously recorded EEG data were set for one second before and one second after the TMS pulse. Data were processed for artifact removal and cortical source localization, followed by energy landscape analysis to extract signatures that characterize physiological brain network mechanisms. The analysis steps are summarized in Figure 1, and discussed in further details in the subsequent subsections.

### 2.1. EEG Data Recording and Preprocessing to ERP

EEG signal data were recorded from 11 scalp cortical sites via Ag/AgCl sintered electrodes cap. The electrodes were F3, FZ, F4, T3, CZ, T4, P3, PZ, P4, O1, and O2 according to the extended 10–20 system with an impedance below 5 kΩ. The ground electrode was placed on the forehead and a nose electrode served as a reference. TMS subthreshold pulse was delivered over each cortical region of each participant through a figure-of-eight coil (70 mm outer diameter of each wing) using Magstim 200 Magnetic stimulators with a monophasic current waveform (Magstim Co., Whitland, UK) [21]. The cortical site(s) at which we administered the TMS subthreshold pulse along with the count of patients and controls were the left motor cortex, prefrontal cortex, and the posterior cerebellum as summarized in Table 1. Our subjects aged between 18 to 62 years (40 male and 22 female) with a criterion as described in Table 2.

The offline analysis was conducted by using Neuroscan 4.3 software for eyeblink artifact removal: MATLAB (MathWorks, Inc., Natick, MA, USA)-based EEGLAB [30], and python-based Neuropype (https://www.neuropype.io (accessed on 13 April 2023)) for EEG artifact removal and processing to ERP-based source localization. For this, EEG data were set 1000 ms prior and after the TMS pulse, followed by ±75 µV thresholding and a bandpass filter (1 to 50 Hz), and then they averaged to event-related potential (ERP) for increased SNR as shown in Equation (1).

(1) $$x^{\prime }\left(t\right)=\frac{1}{N}\overset{N}{\underset{k=1}{\sum }}x\left(t,k\right)=s\left(t\right)+\frac{1}{N}\overset{N}{\underset{k=1}{\sum }}n\left(t,k\right)$$

where $x^{\prime }\left(t\right)$ = average of N trials, i.e., ERP signal that is hoped to be $s\left(t\right)$

$s\left(t\right)$ = signal
$n\left(t\right)$ = noise in the signal data
N = number of trials, roughly 60 on average across subjects
t = time elapsed after the kth event

**Figure 2:**
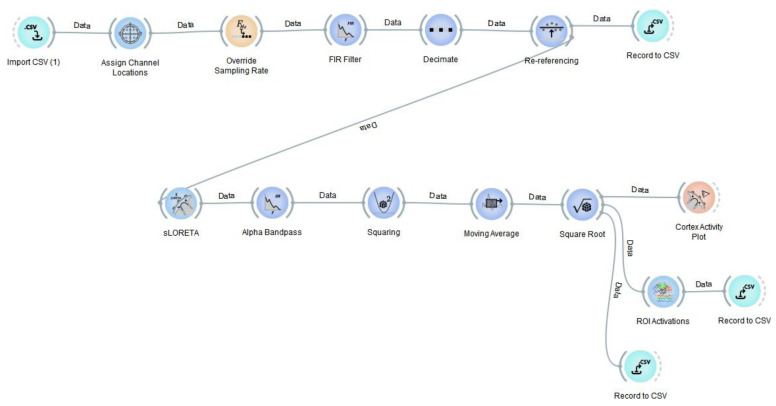
We used python-based Neuropype pipeline to process 2D scalp ERP data, which is extracted from EEG data after artifact removal, for source localization. The obtained current density estimates of voxels belonging to the same region is averaged for getting ROI activations for determining source localization estimates in a CSV output format.

As shown in Figure 2, in the processing pipeline, the imported preprocessed ERP signal data were first FIR bandpass filtered (1–50 Hz), then decimated, re-referenced with all channels’ mean as the referral, and source localized by using the sLORETA algorithm. Decimation aids computational load by reduction factor of 2 (i.e., 2000 ms of ERP signal data down samples to 1000 ms data segment). The pipeline then applies an alpha brainwave bandpass filter (8–12 Hz) and retrieves power current density value estimates for the alpha brain wave activity of the voxels. We used the sLORETA-algorithm-based Neuropype pipeline for source localization of preprocessed EEG data.

### 2.2. Cortical Source Localization of ERP

The sLORETA, abbreviated for standardized low-resolution brain electromagnetic tomography, is a well-established and accepted technique to estimate source current density inside the brain for the given electroencephalographical data by solving the inverse problem [31] as shown in Equation (2), at a spatial resolution of 5 mm based on the digitized Talairach atlas provided by the Brain Imaging Centre Montreal Neurological Institute [32] and is widely accepted.

(2) ϕ=KJ+c1

where ϕ = scalp electric potentials matrix ∈ R^N_E_^^×1^ where N_E_ is number of electrodes

K = lead field matrix ∈ R^N_E_×3N_V_^
J = primary current density matrix ∈ R^3N_V_^^×1^
c = arbitrary constant which embodies the fact that electric potential is determined up to an arbitrary constant

The more the number of electrodes used, the higher the source localization accuracy. We recognize that 11 electrodes may not give the best source localization outcome. However, based on the work of Pascual et al., we assume minimized error, i.e., zero error localization because in all noisy simulations. sLORETA has by far the lowest localization errors as compared with minimum norm such as Dale et al. and so on [31]. In most cases, the spatial spread (i.e., “blurring”) of sLORETA is smaller than that of the Dale method [31,33]. Future studies with a larger number of scalp electrodes may consider our study with 11 electrodes as a baseline work for when they get higher resolution estimations for signature validation and comparison.

The obtained current densities’ estimations of voxels belonging to the same brain region were averaged together to obtain that region’s total current density. Brain regions belonging to the same functional network, as was per the fMRI atlas (neurosynth; https://neurosynth.org/locations/ (accessed on 13 April 2023)) [34,35], were then taken for network data-based energy landscape analysis. Next, brain network energy landscape analysis was performed to retrieve stable network’s energies as discussed in the subsequent section. These brain network energies distinguish patients from controls.

### 2.3. Localized Functional Brain Network Energy Landscape Analysis

Two duration of network constituent regions’ signal data activity are taken, i.e., 1–500 sample values of current density for 1 second of pre-TMS pulse signal data, and 501–1000 current density sample values for post-TMS pulse signal data. For each duration, average signal data were computed, and set as the threshold for binarization (value > threshold value is 1 for active, otherwise −1 for inactive). Considering that a region can be in 2 states; either active or inactive, and a total of M number of constituent regions exist, then a network’s activity pattern at an instance will be defined by a vector with 2^M^ possible states. Finally, relative frequency (P), with which each activity pattern is visited, is calculated as per equation [3]. It is based on the energy (E) of that activity pattern which uses maximum entropy to impose parameter selection (h,j) with respect to empirical and model distribution as also discussed in detail with fMRI signal data by Ezaki et al. [24].

(3) $$P=\exp\left[-E\right]/\sum \exp{\left[-E\right]}^{\prime }$$

where P = relative frequency with which each activity pattern is visited, and E = energy of the activity pattern

As per the energy landscape analysis technique, we first calculated the activity map, i.e., a connectivity state, for group-wise all subjects’ data combined for both pre- and post-TMS condition scenarios along with their corresponding energy dysconnectivity graph for all patients’ data combined and similarly for all controls’ data combined. We then short-select the stable activity pattern which is determined by taking the ones that exhibit least energy values. Next, we calculated individual subject-wise energies for these specific short-selected activity patterns. Here, we considered 7 networks namely the sensorimotor, auditory, default-mode, visual, frontoparietal, salience, and the attention network as summarized in Supplementary Materials (Table S1 and Figure S1) [34,36]. Finally, we performed statistical tests on the obtained energy values to extract biomarkers as discussed in the next section.

### 2.4. Statistical Analysis for Relevant Signature Extraction

To select the signatures that significantly distinguish patients from controls for diagnosis of schizophrenia in clinical applications, statistical 2 sample *t*-test with Bonferroni correction was performed on the brain network energy values. On the obtained energy values for patients and controls, we performed paired 2 sample *t*-test to compare:

1. activity pattern (i.e., brain network state) energy values of controls in pre-TMS condition and post-TMS condition
2. activity pattern energy values of patients in pre-TMS condition and post-TMS condition.
3. activity pattern energy values of group of controls with that of patients during pre-TMS condition.
4. activity pattern energy values of group of controls with that of patients during post-TMS condition

Next, the Bonferroni correction was applied on the *p*-values. This implies that, for each network, if the number of total obtained short-selected activity patterns state was n, then our Bonferroni correction would need to be satisfied where *p*-value < α/n (α=0.05). This yielded 29 signatures and we examined their *p*-values. The signatures that satisfy the least Bonferroni correction (*p*-value ≤ 5 × 10^−5^) were short selected as the most relevant, reliable, and stable signatures out of all the signatures obtained. By selecting this Bonferroni corrected *p*-values threshold value at 5 × 10^−5^, we emphasize large effect size (average Cohen’s d value = 1.22), thereby giving us significant signatures.

## 3. Results

Six out of the 29 brain connectivity network signatures were selected based on the *p*-value that satisfies Bonferroni correction, to distinguish schizophrenia patients from controls. These signatures, shown in Table 3, Table 4 and Table 5, were obtained from our novel method pipeline described in the methods section earlier.

In the table, first two columns list the constituent regions of the network along with its active status (−1 implies inactivity whereas 1 implies active and good connectivity to other constituent regions). Together these two columns represent the network’s state. The third column explains the cognitive and physiological significance of the network state. The fourth column lists which region was stimulated that triggered the state and the conditions that differentiate the groups for that stimulus (pre- and post-TMS of controls or patients). Finally, the fifth column provides the *p*-value that satisfies the Bonferroni correction.

Thus, the tables in the description represent the results of a study that aimed to assess the functional connectivity of brain regions. The columns in the tables list information about the state of the network, its cognitive implications, the stimulus that triggered the state, and the statistical significance of the results. These 29 brain network signatures are listed in Section 3.1, and the six most relevant reliable signatures are described in more detail in Section 3.2.

### 3.1. All Localized Brain Network Signatures

**Figure 3:**
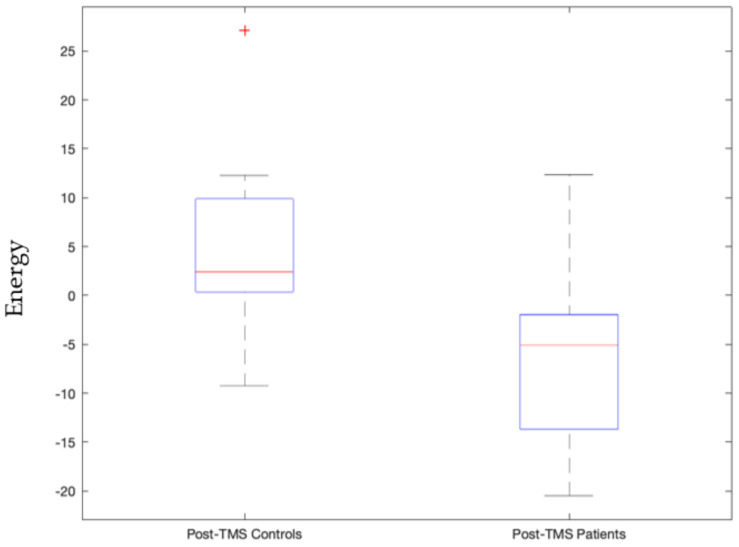
Energy values of controls significantly differed from patients during post-TMS subthreshold pulse stimulation condition with a *p*-value = 0.00003 for the sensorimotor network energy state where left precentral and postcentral gyrus along with paracentral lobule are actively bonded together in terms of connectivity. TMS subthreshold pulse was administered at the left motor cortex.

Upon stimulating the left motor cortex, we found a sensorimotor network state, where left precentral and postcentral gyrus along with paracentral lobule are actively bonded together in terms of connectivity, i.e., their current densities are above the average threshold value whereas the right hemisphere’s precentral and postcentral gyrus are inactive. Given the contralateral nature of control, this state correlates to the motion in the right side of the body. This stable sensorimotor network state’s pre-TMS pulse condition was significantly different from post-TMS pulse condition in controls as shown in Supplementary Material Figure S2 (*p*-value = 0.0000055) whereas for patients this was not the case. Moreover, this state’s energy also significantly differed post-TMS pulse condition in patients than controls as shown in Figure 3 (*p*-value = 0.00003). Additionally, when the left prefrontal cortex is stimulated, this sensorimotor network state is invoked in the case of which post-TMS conditions of patients and controls significantly differed (*p*-value = 0.0059).

Inactive state of sensorimotor network is observed on vermis’ stimulation in the case of which patients significantly differ in their pre- and post-TMS pulse condition as well as patients and controls significantly differ post-TMS pulse condition. This state signifies moments when the subject is not actively performing or processing any sensory input or motor activity, all constituent regions’ current densities are below the average threshold value, and it becomes a stable active state where pre-TMS condition was significantly different from post-TMS pulse condition in controls as shown in Supplementary Material Figure S3 (*p*-value = 0.0018) whereas for patients this was not the case. Moreover, this state’s energy also significantly differed in patients than controls post-TMS pulse condition as shown in Supplementary Material Figure S4 (*p*-value = 0.002). Interestingly, this seems to be a similar case as when the left motor cortex is stimulated thereby indicating that the Vermis may impact or is well connected with the motor cortex.

We also observe that two stable energy biomarker states of the visual cortical network get invoked when the left motor cortex is stimulated by a TMS subthreshold pulse. The first state is defined when all constituent regions of the visual network are inactive, i.e., below activation threshold value. This state’s energies significantly distinguish pre- and post-TMS condition of controls (*p*-value = 0.0016) as well as of patients (*p*-value = 0.00032), and it also distinguishes patients from controls in both pre- and post-TMS subthreshold pulse condition (*p*-value = 0.0016 and *p*-value = 0.0083) as shown in Supplementary Material Figure S5.

**Figure 4:**
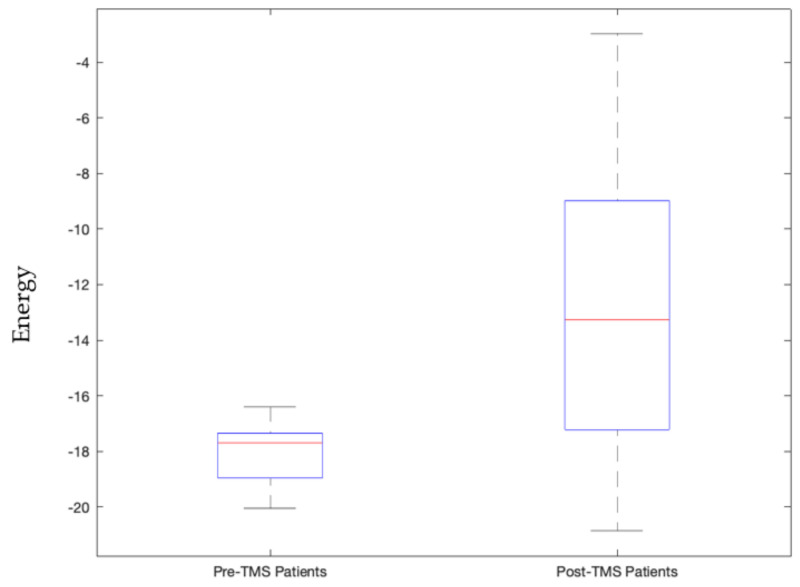
Energy values of patients during pre-TMS subthreshold pulse stimulation condition significantly differed from their post-TMS subthreshold pulse stimulation condition with a *p*-value = 0.000036 for the active visual network energy state. TMS subthreshold pulse was administered at the left motor cortex.

When the visual network is in an active state, i.e., its constituent regions, namely, Pericalcarine Gyrus, Lingual Gyrus, Lateral Occipital Gyrus, Fusiform Gyrus, and the Cuneus Gyrus, are actively connected together such that their current densities are above threshold value, then patients’ energies significantly differed in their pre- and post-TMS pulse conditions (*p*-value = 0.000036) as shown in Figure 4. For controls, this was not the case. Furthermore, post-TMS pulse condition of patients was significantly different from controls during this active visual network state (*p*-value = 0.0035) as shown in Supplementary Material Figure S6.

Additionally, on left motor cortex stimulation, we see when there is an active frontoparietal network energy state where if its constituent regions (left and right) Superior Frontal Gyrus, (rostral and caudal) Middle Frontal Gyrus, Pars Triangularis Gyrus, Medial Orbitofrontal Gyrus, and Inferior Parietal Gyrus are above threshold current densities i.e., actively bonded then pre and post-TMS pulse condition energies in patient (*p*-value = 0.0011) are significantly different as can be seen in Supplementary Material Figure S7, whereas in controls, that is not the case.

**Figure 5:**
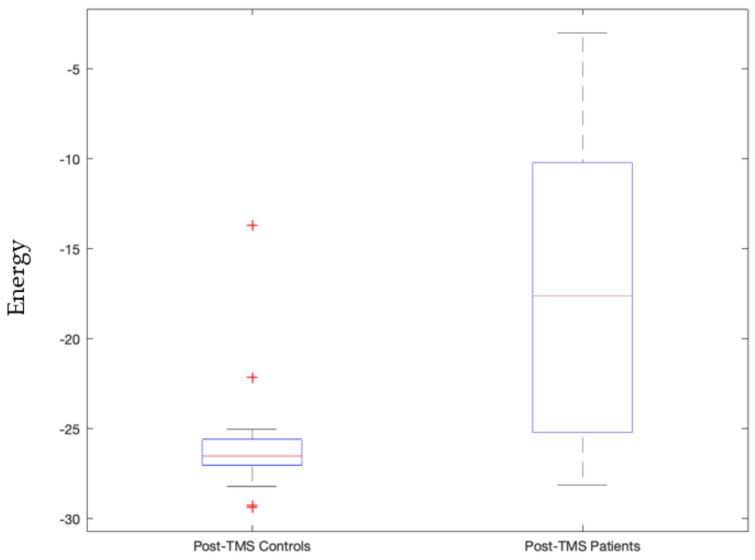
Energy values of controls significantly differed from patients during post-TMS subthreshold pulse stimulation condition at the vermis with a *p*-value = 0.000048 for the inactive energy state of the frontoparietal network. TMS subthreshold pulse was administered at the vermis.

We also see that when the frontoparietal network is entirely inactive then on TMS subthreshold pulse stimulation at the Vermis, patients’ pre- and post-TMS pulse condition is significantly different (*p*-value = 0.00083) as shown in Supplementary Material Figure S8. Furthermore, controls and patients were significantly distinguishable in post-TMS pulse condition in this inactive frontoparietal network (*p*-value = 0.000048) as shown in Figure 5.

**Figure 6:**
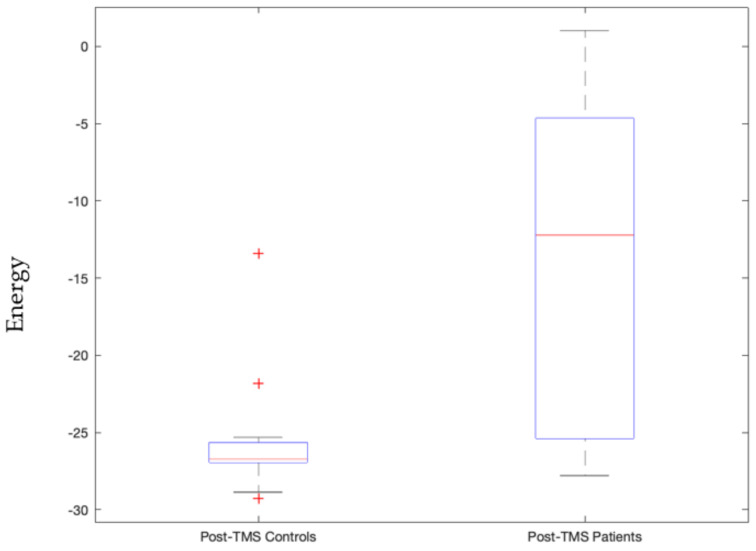
Energy values of controls significantly differed from patients during post-TMS subthreshold pulse stimulation condition at the vermis with a *p*-value = 0.0000099 for the active energy state of the frontoparietal network.

Other than that, when the frontoparietal network is in an active state, then patients pre-TMS condition were significantly different from post-TMS pulse condition (*p*-value = 0.0004), as shown in Supplementary Material Figure S9. This same active frontoparietal network state also significantly distinguished patients from controls in post-TMS subthreshold pulse condition (*p*-value = 0.0000099) as shown in Figure 6.

**Figure 7:**
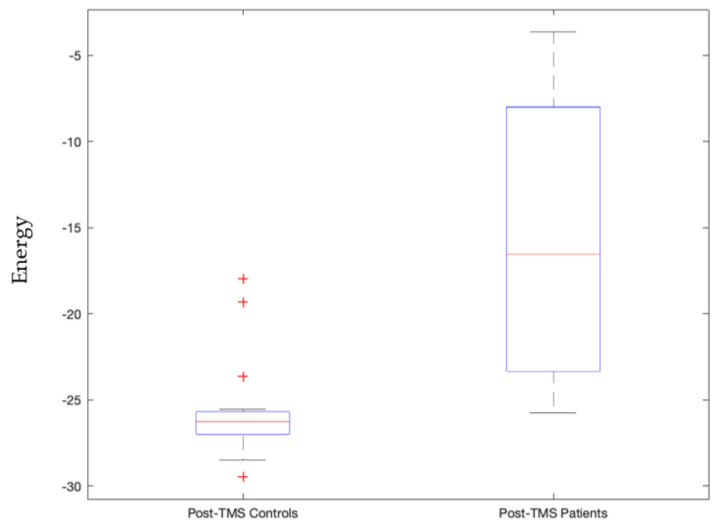
Energy values of controls significantly differed from patients during post-TMS subthreshold pulse stimulation condition with a *p*-value = 0.00014 for the inactive salience network energy state. TMS subthreshold pulse was administered at the vermis.

**Figure 8:**
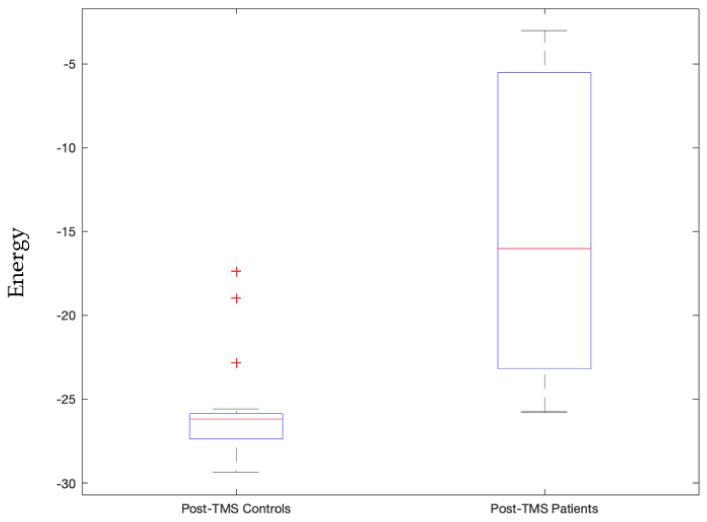
Energy values of controls significantly differed from patients during post-TMS subthreshold pulse stimulation condition with a *p*-value = 0.0000015 for the active salience network energy state. TMS subthreshold pulse was administered at the vermis.

Another connectivity network state was found to be when the salience network was entirely inactive. Patients’ pre- and post-TMS pulse condition was significantly different (*p*-value = 0.00014) as shown in Supplementary Material Figure S10 whereas for controls this was not the case. It also significantly distinguishes patients from controls in their post-TMS condition (*p*-value = 0.0000014) as shown in Figure 7. The same is true when the salience network is completely active. Pre- and post-TMS condition are significantly different in patients (*p*-value = 0.00012) and post-TMS significantly differs from that of controls (*p*-value = 0.0000015). This is shown in Supplementary Material Figure S11 and Figure 8 respectively.

A few other signatures include scenarios with the attention network as summarized in Table 5. For example, on stimulating the left motor cortex with the TMS subthreshold pulse, energy states in the attention network where when constituent regions (right and left) superior temporal gyrus, superior parietal gyrus, pars triangularis gyrus, inferior parietal gyrus are actively connected thereby excluding just the rostral and caudal middle frontal gyrus then post-TMS pulse condition of controls, and patients are observed to be significantly different (*p*-value = 0.0022). This state is also invoked when the left prefrontal cortex is stimulated, which again significantly distinguishes patients from controls post-TMS (*p*-value = 0.0023), and when the vermis is stimulated that significantly distinguishes pre and post-TMS pulse condition of patients (*p*-value = 0.0042). Similarly, for further signature scenarios of the attention network and the auditory network, the reader is encouraged to refer to Table 3 and Table 5 respectively. We now discuss the most relevant signatures out of these in the next subsequent sections.

### 3.2. Most Relevant Reliable Brain Signatures

We view signatures that satisfy Bonferroni-corrected *p*-values, with 5 × 10^−5^ threshold, as the most trustworthy and relevant, as this indicates statistically high significance for that signature. This includes the following stable signatures:

Signature 1. Left motor cortex stimulation invoked sensorimotor network state that governs the right side of the body significantly distinguishes between patients and controls post-TMS condition (*p*-value = 0.00003, Cohen d’s value = 0.85) and pre- and post-TMS pulse condition of controls and not patients (*p*-value = 0.0000055). The latter validates our earlier study [42].
Signature 2. Left motor cortex stimulation invoked visual network active state that significantly distinguished pre- and post-TMS pulse condition of patients (*p*-value = 0.000036, Cohen d’s value = 0.64). This state governs times when the subject is processing ocular information or spatial awareness [36].
Signature 3. Vermis stimulation invoked frontoparietal network’s inactive state that significantly distinguished post-TMS pulse condition of patients from that of controls (*p*-value = 0.000048, Cohen d’s value = 1.09). This inactive frontoparietal state implies that the subject is not involved in the thought processes related to decision making or problem solving.
Signature 4. Vermis stimulation invoked frontoparietal network’s active state that significantly distinguished post-TMS pulse condition of patients from that of controls (*p*-value = 0.0000099, Cohen d’s value = 1.85). This state governs thought processes related to decision making or problem solving.
Signature 5. Vermis stimulation invoked salience network’s inactive state that significantly distinguished post-TMS pulse condition of controls and patients (*p*-value = 0.00014, Cohen d’s value = 1.38). This state implies that the subject is not in a decision-making process of attention to the environment stimulus.
Signature 6. Vermis stimulation invoked salience network’s active state that significantly distinguished patients and controls’ post-TMS conditions (*p*-value = 0.0000015, Cohen d’s value = 1.53). This state is also stably invoked where when the subjects are not invested in deciding their attention for the environmental stimulus.

In summary, left motor cortex invokes sensorimotor cortex governing the right side of the body and stimulation to the vermis results in inducing highest number of connectivity network signatures.

## 4. Discussion

The goal of our study is to use TME-EEG signals to extract connectivity signatures that can be used to separate control and patients. As shown in Figure 1, Figure 5, Figure 6, Figure 7 and Figure 8, for each of the connectivity signatures the control group has either higher or lower average energies, which means weaker or stronger connectivity respectively among these corresponding brain networks or regions. Using multi-site TMS coils to simultaneously or sequentially activate these brain regions [22,43,44,45,46], we can modify the connectivity among these regions. The developed measurement method can be used to quantitatively study connectivity signatures before and after multisite stimulations. Thus, these connectivity signatures can be used to monitor treatment effects.

From our analysis, we saw largest number of signatures in response to the vermis subthreshold TMS stimulation involving inactive and active state of both frontoparietal and salience network. This conveys that the vermis is more densely connected to all the other networks in the brain than any other regions. Additionally, we observe that when the left motor cortex is stimulated by the subthreshold TMS, the sensorimotor brain network that governs the contralateral right side of our body, significantly distinguishes the schizophrenia patients from healthy individuals. Lastly, the TMS to prefrontal cortex invokes sensorimotor and attention network signatures that distinguish patients and controls, though with relatively lesser significant magnitude, than left motor cortical and vermis stimulations. These signatures as stated in the results section are also further explained below:

Signature 1. This sensorimotor network state, invoked by left motor cortex stimulation, which significantly distinguishes between patients and controls post-TMS condition, is comprised of three active constituents, namely the pre- and postcentral gyrus in the left hemisphere, and two inactive constituents, namely the pre- and post-central gyrus in the right hemisphere. This state governs the right side of the body given we know the contralateral control based nature of the sensorimotor network [47]. As can be seen in Figure 3, this implies that the left precentral and postcentral gyrus along with paracentral lobule are actively bonded together in terms of connectivity and the control group has higher energy levels (weaker connectivity) than those of the patients. The implication is that it may be good to have weaker connectivity among these regions.
Signature 2. The active visual network state, where all constituent regions of the visual network are active, invoked by left motor cortex stimulation that significantly distinguished pre- and post-TMS pulse condition of patients, governs instances where the subject is processing ocular information or spatial awareness [36]. It can be seen in Figure 4 that this state’s energy values are lower during the pre-TMS condition than that during the post-TMS condition in patients, implying that after TMS the connectivity among these regions gets weaker in patients.
Signature 3. The frontoparietal network’s inactive state, invoked by vermis stimulation, that significantly distinguishes post-TMS pulse condition of patients from that of controls, including all constituents in the inactive state. This inactive frontoparietal state implies that the subject is not involved in the thought processes related to decision making or problem solving [48]. As can be seen in Figure 5, energy values of this state are higher than that in the patients, thereby implying that the constituent regions are more connected in controls than in patients during post-TMS condition.
Signature 4. The frontoparietal network’s active state, invoked by vermis’ stimulation, significantly distinguished post-TMS pulse condition of patients from that of controls. This state governs thought processes related to decision making or problem solving [48]. As can be seen in Figure 6, energy values of this state are higher than those in the patients, thereby implying that the constituent regions are more connected in controls than in patients during post-TMS condition.
Signature 5. This salience network’s inactive state, invoked by the vermis stimulation, that significantly distinguished post-TMS pulse condition of controls and patients, implies that the subject is not in a decision-making process of attention to the environment stimulus [49]. As can be seen in Figure 7, energy values of this state are higher than those in the patients, thereby implying that the constituent regions are more connected in controls than in patients during post-TMS condition.
Signature 6. Vermis stimulation invoked the salience network’s active state that significantly distinguished patients’ and controls’ post-TMS conditions. This state is also stably invoked when the subjects are not invested in deciding their attention for the environmental stimulus [49]. As can be seen in Figure 8, energy values of this state are higher than those in the patients, thereby implying that the constituent regions are more connected in controls than in patients during post-TMS condition.

These extracted signatures will need to be further studied by validating their correlation with other patient neurophysiology test results. This pilot study is a resting-state study and done with a relatively lower number of channels. Future studies will include a higher number of electrodes with subjects executing cognitive tasks and with additional disorder symptom measurements for further analysis.

Furthermore, our novel data driven method pipeline estimates brain network energies, in response to subthreshold TMS stimulation to three different stimulation sites—left motor cortex, left prefrontal cortex and the vermis—on a clinical schizophrenia population, by using sLORETA as the source localization algorithm with lowest localization error as compared to other localization methods [31]. We selected and stated 6 out of the 29 signatures yielded by our method that significantly distinguished patients from controls.

The higher the number of electrodes used, the higher the source localization accuracy. We recognize that 11 electrodes limit the best outcome for source localization. However, based on the work of Pascual et al., we can assume a minimized error because in all noisy simulations, sLORETA has by far the lowest localization errors as compared with minimum norm, Dale, and so on [31,33]. Hence, this TMS-EEG-based energy landscape analysis method we developed here provides a baseline work for future studies with a larger number of scalp electrodes for signature validation and comparison at higher resolution. It may offer a faster, less expensive, and possibly real-time approach for treatment monitoring compared with using the functional magnetic resonance imaging approach [50,51].

## 5. Conclusions

We have conducted the first high temporal resolution source-localized EEG brain network energy study in response to three cortical TMS stimulation sites, namely, the vermis, left motor, and prefrontal cortex with the lowest localization error. Six significant signatures were reported in results and overall connectivity is discussed by using our proposed novel EEG processing pipeline. This includes but is not limited to sensorimotor cortical network signature in response to left motor cortex stimulation and high connectivity response to vermis stimulation. For higher number EEG scalp electrode-associated studies or even other neuroimaging modalities, our work serves as a preliminary baseline to validate and compare brain signature estimations for aiding clinical applications with brain connectivity signature extraction. The developed TMS-EEG connectivity measurement method may provide a faster, less expensive, and possibly real-time approach for treatment monitoring.

## Figures and Tables

**Table 1:** Summary of subjects counted for gathered EEG data from three stimulated cortical sites by TMS subthreshold pulse.

Stimulated Cortical Site	Schizophrenia Patients	Healthy Controls	Total Subjects
Left motor cortex	22	27	49
Left prefrontal cortex	9	18	27
Vermis	12	25	37

**Table 2:** Summary of the inclusion and exclusion criteria of the human subjects for our study.

Inclusion Criteria:
Male and female between ages 18–62 Ability to give written informed consent (age 18 or above) For patient participants, Evaluation to Sign Consent (ESC) above 10.
**Exclusion Criteria:**
Any history of seizures. Any family history of epilepsy in first-degree relatives. Significant alcohol or other drug use (substance dependence within 6 months or substance abuse within 1 month) other than nicotine or marijuana dependence. Family history of epilepsy in first-degree relatives. Any major medical illnesses that may affect normal brain functioning. Examples of these conditions include, but are not limited to, stroke, CNS infection or tumor, other significant brain neurological conditions. Taking >400 mg clozapine/day Failed TMS screening questionnaire Cardiac pacemakers, implanted medication pumps, intracardiac lines, or acute, unstable cardiac disease, with intracranial implants (e.g., aneurysm clips, shunts, stimulators, cochlear implants, or electrodes) or any other metal object within or near the head, excluding the mouth, that cannot be safely removed. History of head injury with loss of consciousness over 10 min; history of brain surgery Cannot refrain from using alcohol and/or marijuana 24 h or more and cigarette smoking half an hour or more prior to experiments. A woman who is pregnant (child-bearing potential but not on contraceptive and missing menstrual period; or by self-report; or by positive pregnancy test)

**Table 3:** Connectivity network state summary for the states of sensorimotor, visual, and auditory network when the left motor, left prefrontal cortex, and vermis are stimulated by the TMS subthreshold pulse.

Invoked brain network state with the list of constituent regions and status (1 = active, −1 = inactive)				
Sensorimotor network state:		Network state’s cognitive definition	Stimulated regions and observed significant differences	Bonferroni corrected *p*-value
1	Left Precentral Gyrus	This state correlates to the sensorimotor activity in the right side of the body	1. Left Motor Cortex Stimulated:	
1	Left Postcentral Gyrus		a. Significantly distinguished pre and post TMS condition in controls	0.0000055
1	Paracentral Lobule		b. Significantly distinguished patients and controls in post TMS condition	0.00003
−1	Right Precentral Gyrus		2. Left Prefrontal Cortex Stimulated:	
−1	Right Postcentral Gyrus		a. Significantly distinguished patients and controls in post TMS condition	0.0059
−1	Left Precentral Gyrus	Subject is not actively performing any sensorimotor activity in this state. His/her behavior excludes any motor response	1. Vermis Stimulated:	
−1	Left Postcentral Gyrus		a. Significantly distinguished pre and post TMS condition in patients	0.0018
−1	Paracentral Lobule		b. Significantly distinguished patients and controls in post TMS condition	0.002
−1	Right Precentral Gyrus			
−1	Right Postcentral Gyrus			
Visual Network inactive and active states:				
−1	Pericalcarine Gyrus	Brain is not focused on processing any infor-mation related to any ocular input and spatial awareness or moving objects in this inactive visual network state	1. Left Motor Cortex Stimulated:	
−1	Lingual Gyrus		a. Significantly distinguished pre and post TMS condition in patients	0.0003278669
−1	Lateral Occipital Gyrus		b. Significantly distinguished pre and post TMS condition in controls	0.0016
−1	Fusiform Gyrus		c. Significantly distinguished patients and controls in post TMS condition	0.0017
−1	Cuneus Gyrus		d. Significantly distinguished patients and controls in pre TMS condition	0.0084
1	Pericalcarine Gyrus	Brain is processing information related to any ocular input and spatial awareness or moving objects in this active visual network state	1. Left Motor Cortex Stimulated:	
1	Lingual Gyrus		a. Significantly distinguished pre and post TMS condition in patients	0.00003623395
1	Lateral Occipital Gyrus		b. Significantly distinguished patients and controls in post TMS condition	0.003594008
1	Fusiform Gyrus			
1	Cuneus Gyrus			
Invoked brain network state with the list of constituent regions and status (1 = active, −1 = inactive)				
Auditory Network state(s):				
1	Left Supramarginal Gyrus	This state involves phonological pro-cessing for egocentric emotional projec-tion in social judgement	1. Vermis Stimulated:	
1	Left Superior Temporal Gyrus		a. Significantly distinguished patients and controls in post TMS condition	0.0028
−1	Right Supramarginal Gyrus			
−1	Right Superior Temporal Gyrus			
−1	Transverse Temporal Gyrus			
−1	Left Supramarginal Gyrus	Brain is processing the incoming auditory information	1. Vermis Stimulated:	
−1	Left Superior Temporal Gyrus		a. Significantly distinguished patients and controls in post TMS condition	0.0033
1	Right Supramarginal Gyrus			
1	Right Superior Temporal Gyrus			
1	Transverse Temporal Gyrus			

Superior Temporal Gyrus—an overview|ScienceDirect Topics. overcoming biased empathic judgments is associated with increased activation in the right supramarginal gyrus (rSMG). source: [37]. extra: [38].

**Table 4:** Connectivity network state summary for the states of frontoparietal, salience, and attention network when the left motor, left prefrontal cortex, and vermis are stimulated by the TMS subthreshold pulse.

Invoked brain network state with the list of constituent regions and status (1 = active, −1 = inactive)				
Frontoparietal Network inactive and active states:		Network state’s cognitive definition	Stimulated regions and observed significant differences	Bonferroni corrected *p*-value
−1	Left Superior Frontal Gyrus	Goal-oriented, problem-solving ability network is inactive. Note: Disruption with nodes of this network leads to schizophrenia (source: Menon, Vinod. “Large-scale brain networks and psychopathology: a unifying triple network model.” Trends in cognitive sciences 15.10 (2011): 483–506.)	1. Vermis Stimulated:	
−1	Right Superior Frontal Gyrus		a. Significantly distinguished pre and post TMS condition in patients	0.0008351
−1	Rostral Middle Frontal Gyrus		b. Significantly distinguished patients and controls in post TMS condition	0.00004847665
−1	Caudal Middle Frontal Gyrus			
−1	Pars Triangularis Gyrus			
−1	Medial Orbitofrontal Gyrus			
−1	Inferior Parietal Gyrus			
1	Left Superior Frontal Gyrus	Goal-oriented, problem-solving ability network is active. Note: Disruption with nodes of this network leads to schizophrenia (source: Menon, Vinod [39]	1. Left Motor Cortex Stimulated:	
1	Right Superior Frontal Gyrus		a. Significantly distinguished pre and post TMS condition in patients	0.001199904
1	Rostral Middle Frontal Gyrus		2. Vermis Stimulated:	
1	Caudal Middle Frontal Gyrus		a. Significantly distinguished pre and post TMS condition in patients	0.0004093212
1	Pars Triangularis Gyrus		b. Significantly distinguished patients and controls in post TMS condition	0.000009929137
1	Medial Orbitofrontal Gyrus			
1	Inferior Parietal Gyrus			
Invoked brain network state with the list of constitutent regions and status (1 = active, −1 = inactive)				
Salience Network inactive and active states:				
−1	Caudal Anterior Cingulate Gyrus	Brain’s salience network state is inactive i.e., it’s not processing information related to learning, decision, survival skills or processing information to switch between the default mode and the frontoparietal network	1. Vermis Stimulated:	
−1	Caudal Middle Frontal Gyrus		a. Significantly distinguished pre and post TMS condition in patients	0.0001468012
−1	Insula Gyrus		b. Significantly distinguished patients and controls in post TMS condition	0.000001478416
−1	Pars Triangularis Gyrus			
−1	Rostral Anterior Cingulate Gyrus			
−1	Rostral Middle Frontal Gyrus			
−1	Superior Parietal Gyrus			
1	Caudal Anterior Cingulate Gyrus	Brain’s salience network state is active i.e., it’s processing information related to learning, decision, survival skills or processing information to switch between the default mode and the frontoparietal network	1. Vermis Stimulated:	
1	Caudal Middle Frontal Gyrus		a. Significantly distinguished pre and post TMS condition in patients	0.0001282761
1	Insula Gyrus		b. Significantly distinguished patients and controls in post TMS condition	0.00000152735
1	Pars Triangularis Gyrus			
1	Rostral Anterior Cingulate Gyrus			
1	Rostral Middle Frontal Gyrus			
1	Superior Parietal Gyrus			

**Table 5:** Connectivity network state summary for the states of the attention network when the left motor, left prefrontal cortex and vermis are stimulated by the TMS subthreshold pulse.

Invoked brain network state with the list of constitutent regions and status (1 = active, −1 = inactive)				
Attention network states:				
1	Right Superior Temporal Gyrus	This state is involved with exogenous or/and endogenous attentive factors related cognitive thinking/behavior (Role of middle frontal gyrus is to converge the attention network to switch attention between endogenous and exogenous factors [40]	1. Left Motor Cortex Stimulated:	
1	Left Superior Temporal Gyrus		a. Significantly distinguished patients and controls in post TMS condition	0.002239524
1	Superior Parietal Gyrus		2. Left Prefrontal Cortex Stimulated:	
1	Pars Triangularis Gyrus		a. Significantly distinguished patients and controls in post TMS condition	0.002384859
1	Inferior Parietal Gyrus		3. Vermis stimulated:	
−1	Rostral Middle Frontal Gyrus		a. Significantly distinguished pre and post TMS condition in patients	0.004248247
−1	Caudal Middle Frontal Gyrus			
1	Right Superior Temporal Gyrus	This activity pattern/state leads to convergence of attention network to visuospatial processing (The right Superior Parietal Lobule and neighboring regions in parietal cortex have been consistently associated with a critical role in visuospatial attention. The right superior parietal lobule has high connectivity with the middle frontal gyrus)	1. Left Prefrontal Cortex Stimulated:	
1	Left Superior Temporal Gyrus		a. Significantly distinguished pre and post TMS condition in patients	0.001543006
−1	Superior Parietal Gyrus		b. Significantly distinguished patients and controls in post TMS condition	0.002384859
1	Pars Triangularis Gyrus			
1	Inferior Parietal Gyrus			
−1	Rostral Middle Frontal Gyrus			
−1	Caudal Middle Frontal Gyrus			
−1	Right Superior Temporal Gyrus	Attention network brain state related to language processing (The inferior parietal lobe is used for mental processing, laguage processing In human interactions [41].	1. Vermis stimulated:	
1	Left Superior Temporal Gyrus		a. Significantly distinguished patients and controls in post TMS condition	0.001144871
1	Superior Parietal Gyrus			
−1	Pars Triangularis Gyrus			
1	Inferior Parietal Gyrus			
−1	Rostral Middle Frontal Gyrus			
−1	Caudal Middle Frontal Gyrus			
1	Right Superior Temporal Gyrus	Attention network state related to mental processing attentiveness with human interaction and related language processing as we know that the pars triangularas gyrus is involved with language processing and comprehension. Source: https://psychology.fandom.com/wiki/Triangular_part_of_inferior_frontal_gyrus, (accessed on 15 April 2023)	1. Vermis stimulated:	
−1	Left Superior Temporal Gyrus		a. Significantly distinguished patients and controls in post TMS condition	0.003343118
−1	Superior Parietal Gyrus			
1	Pars Triangularis Gyrus			
−1	Inferior Parietal Gyrus			
1	Rostral Middle Frontal Gyrus			
1	Caudal Middle Frontal Gyrus			

## Data Availability

Not applicable.

## Supplementary Materials

The following supporting information can be downloaded at: https://www.mdpi.com/article/10.3390/s23084078/s1. Table S1: Summary of networks and their respective functionality. (Note: Subcortical and cingulo-opercular networks are not included; Figure S1: Visual of all nine network and their respective constituent regions (for our study, we considered dorsal and ventral attention network as one attention network). Figure S2: Energy values of controls significantly differed between pre and post TMS subthreshold pulse condition with a *p*-value = 0.0000055 for the sensorimotor network energy state where left precentral and postcentral gyrus along with paracentral lobule are actively bonded together in terms of connectivity. Figure S3: Inactive sensorimotor network state significantly distinguishes energies pre and post TMS subthreshold pulse condition of controls when the vermis is stimulated (*p*-value = 0.0018). Figure S4: Inactive sensorimotor network state significantly distinguishes controls and patients post TMS subthreshold pulse condition when the vermis is stimulated (*p*-value = 0.002). Figure S5: Energy of inactive visual network biomarker state in patients and controls during pre and post TMS pulse condition. Figure S6: Active visual network state significantly distinguishes controls and patients post TMS subthreshold pulse condition when the left motor cortex is stimulated (*p*-value = 0.0035). Figure S7: Active frontoparietal network state significantly distinguishes energies pre and post TMS subthreshold pulse condition of patients when the left motor cortex is stimulated. (*p*-value = 0.0012). Figure S8: Inactive frontoparietal network state significantly distinguishes energies pre and post TMS subthreshold pulse condition of patients when the vermis is stimulated (*p*-value = 0.00083). Figure S9: Active frontoparietal network state significantly distinguishes energies pre and post TMS subthreshold pulse condition of patients when the vermis is stimulated (*p*-value = 0.0004). Figure S10: Inactive salience network state significantly distinguishes energies pre and post TMS subthreshold pulse condition of patients when the vermis is stimulated (*p*-value= 0.00014). Figure S11: Active salience network state significantly distinguishes energies pre and post TMS subthreshold pulse condition of patients when the vermis is stimulated (*p*-value = 0.00012).
