# Supplementary material for: Brain Connectivity Signature Extractions from TMS Invoked EEGs
Source: Sensors (Basel). 2023 Apr 18;23(8):4078. doi: 10.3390/s23084078 (PMC10146617; doi:10.3390/s23084078)
Supplement: Supplementary file 1 [file sensors-23-04078-s001.zip › sensors-2290043-supplementary.pdf]

Brain Connectivity Signature Extractions from TMS Invoked EEGs

Deepa Gupta [1,*], Xiaoming Du [2], Ann Summerfelt [2], L. Elliot Hong [2] and Fow-Sen Choa [1]

[1] Computer Science and Electrical Engineering, University of Maryland Baltimore County, 1000 Hilltop Circle, Baltimore, MD 21227, USA
[2] Maryland Psychiatric Research Center, University of Maryland School of Medicine, 655 W. Baltimore Street, Baltimore, MD 21201, USA
* Correspondence: deepag1@umbc.edu

Supplementary Materials

Table S1 Summary of networks and their respective functionality. (Note: Subcortical and cingulo-opercular networks are not included.)[1]

Network	Function of the network
Sensorimotor	Process sensory input from our senses and aid in executing motor activity.
Auditory	Receive and process contents of sound, voices, or music.
Default-mode	Activates during unfocused activity when the person thinks about oneself, remembers the past and envisions the future more than the task being performed.
Visual	Process ocular activity and information about observed moving or static objects for spatial awareness and guidance.
Frontoparietal	Execute goal-oriented or cognition-demanding tasks to solve problems, make decisions and establish memory.
Salience	Integrate and process relevant information and knowledge for decision establishment and execution.
Attention	Controls attention and direction of stimuli.

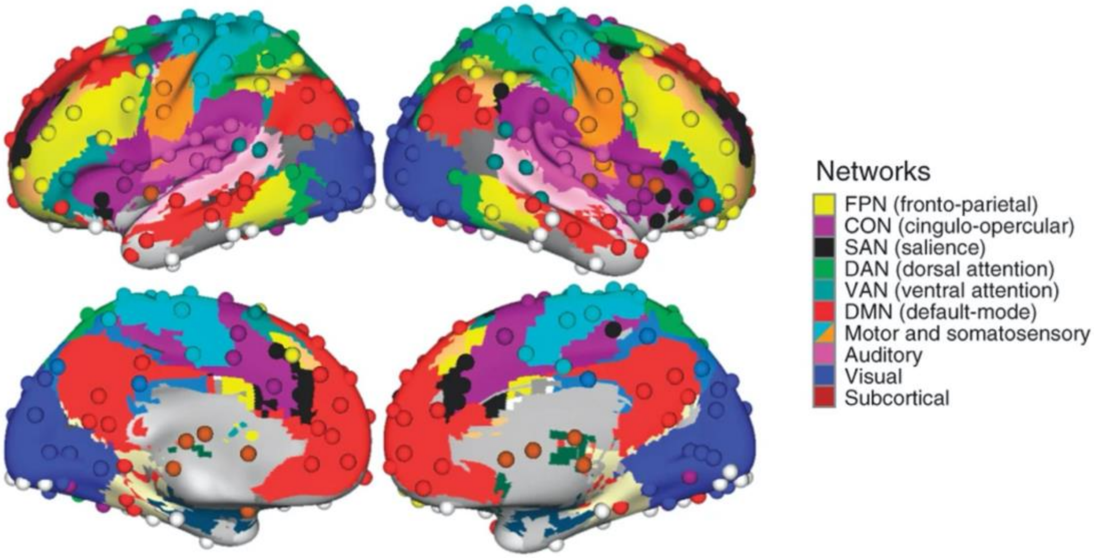

Figure S1 Visual of all nine network and their respective constituent regions (for our study, we considered dorsal and ventral attention network as one attention network)[2]

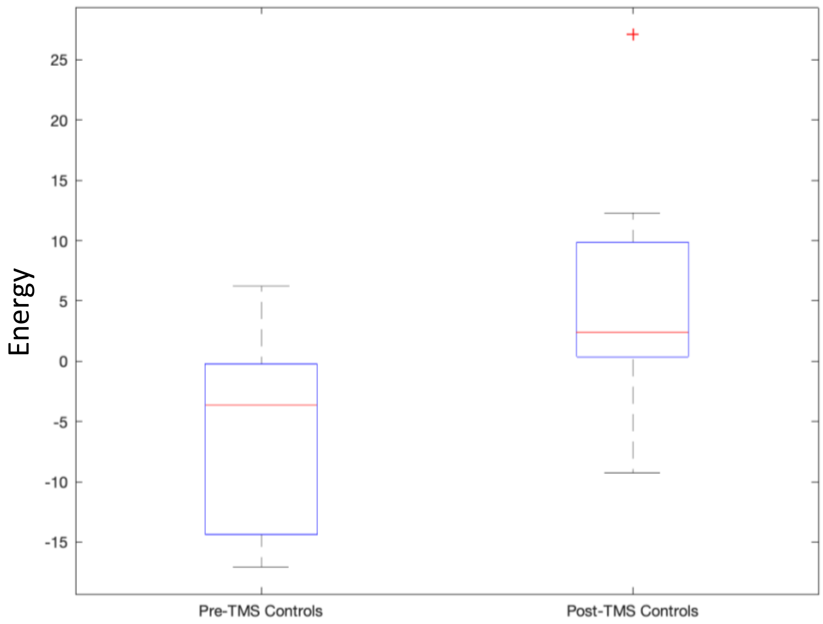

Figure S2 Energy values of controls significantly differed between pre and post TMS subthreshold pulse condition with a p-value=0.0000055 for the sensorimotor network energy state where left precentral and postcentral gyrus along with paracentral lobule are actively bonded together in terms of connectivity

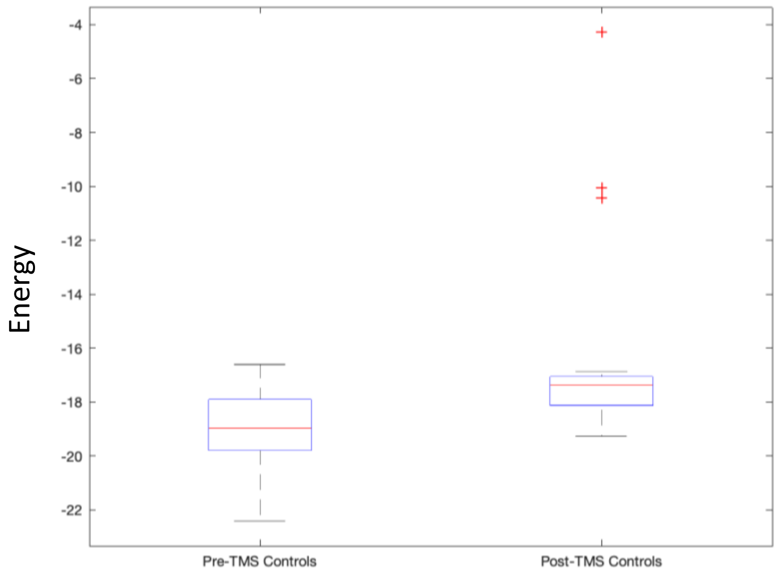

Figure S3 Inactive sensorimotor network state significantly distinguishes energies pre and post TMS subthreshold pulse condition of controls when the vermis is stimulated (p-value=0.0018).

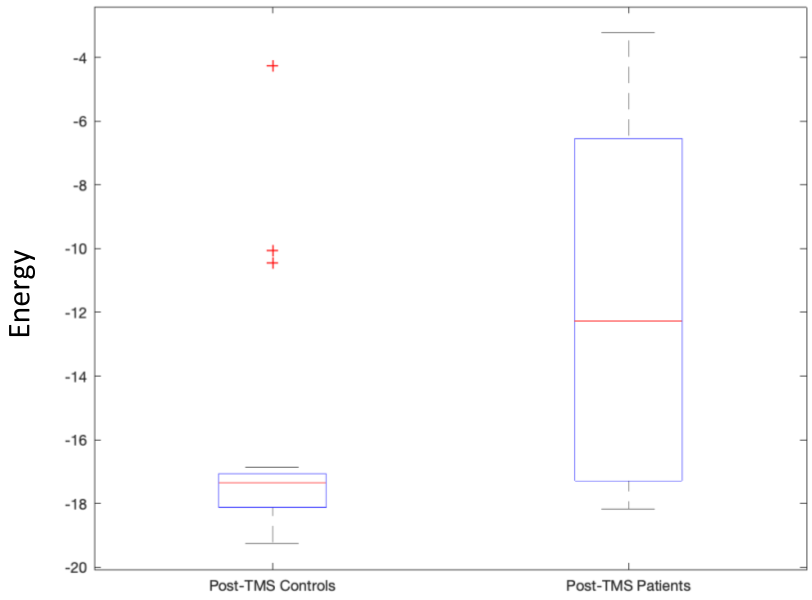

Figure S4 Inactive sensorimotor network state significantly distinguishes controls and patients post TMS subthreshold pulse condition when the vermis is stimulated (p-value= 0.002).

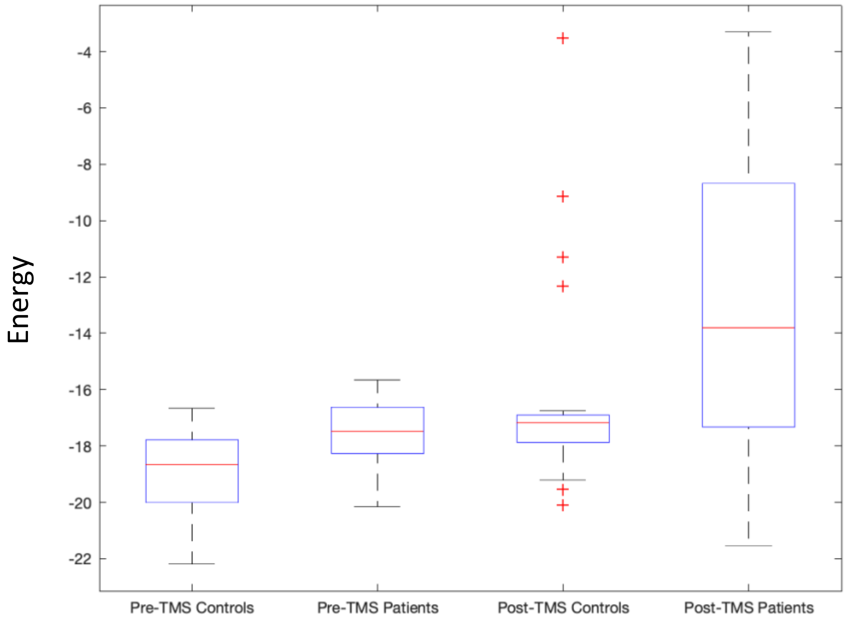

Figure S5 Energy of inactive visual network biomarker state in patients and controls during pre and post TMS pulse condition.

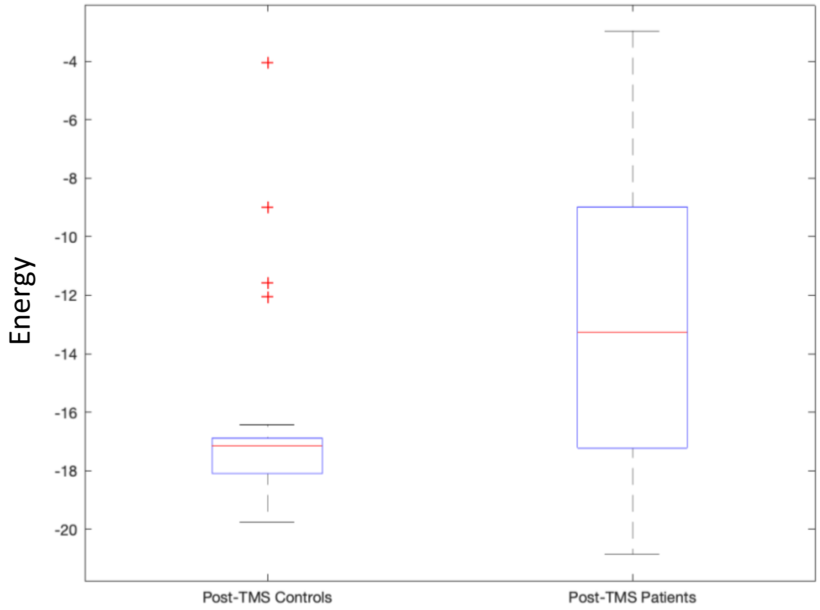

Figure S6 Active visual network state significantly distinguishes controls and patients post TMS subthreshold pulse condition when the left motor cortex is stimulated (p-value = 0.0035).

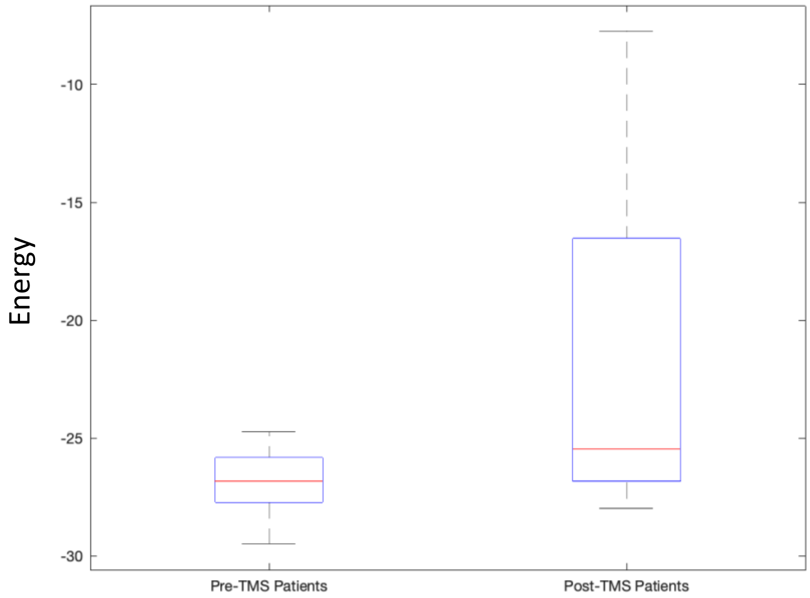

Figure S7 Active frontoparietal network state significantly distinguishes energies pre and post TMS subthreshold pulse condition of patients when the left motor cortex is stimulated. (p-value = 0.0012)

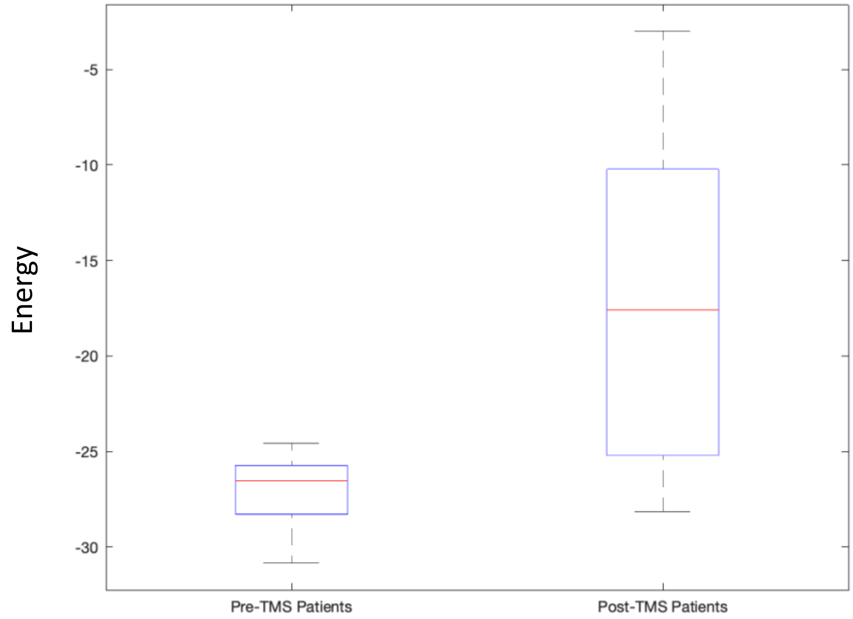

Figure S8 Inactive frontoparietal network state significantly distinguishes energies pre and post TMS subthreshold pulse condition of patients when the vermis is stimulated (p-value= 0.00083).

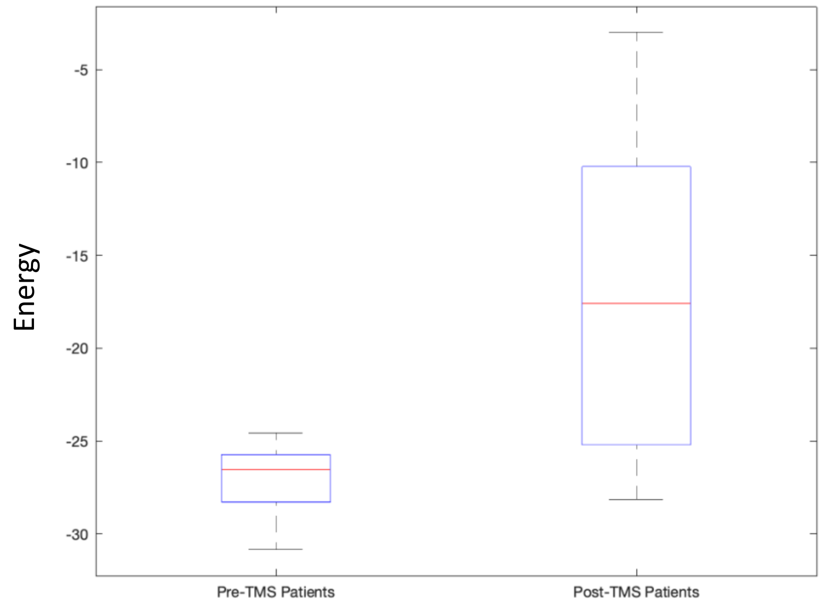

Figure S9 Active frontoparietal network state significantly distinguishes energies pre and post TMS subthreshold pulse condition of patients when the vermis is stimulated (p-value = 0.0004)

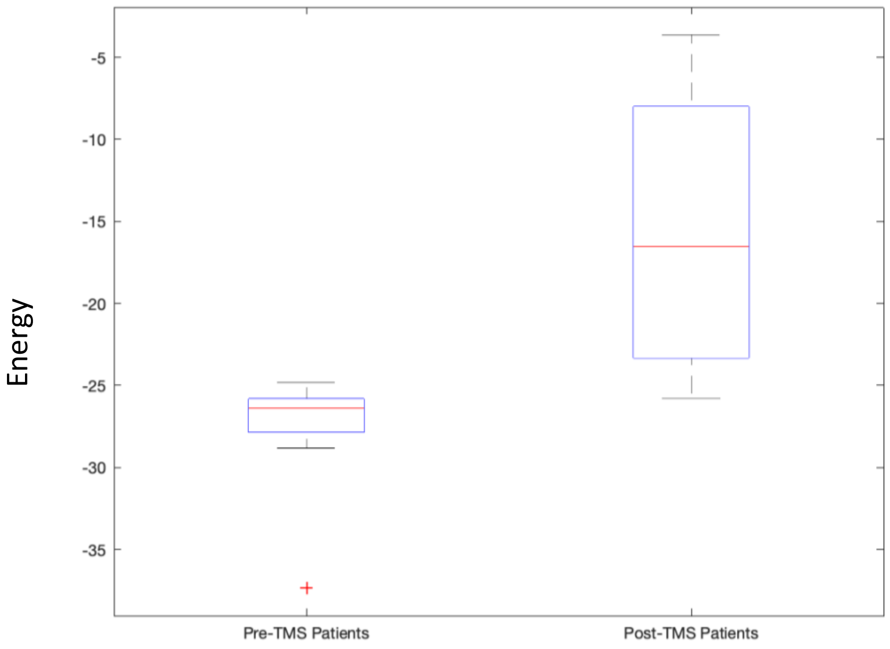

Figure S10 Inactive salience network state significantly distinguishes energies pre and post TMS subthreshold pulse condition of patients when the vermis is stimulated (p-value= 0.00014).

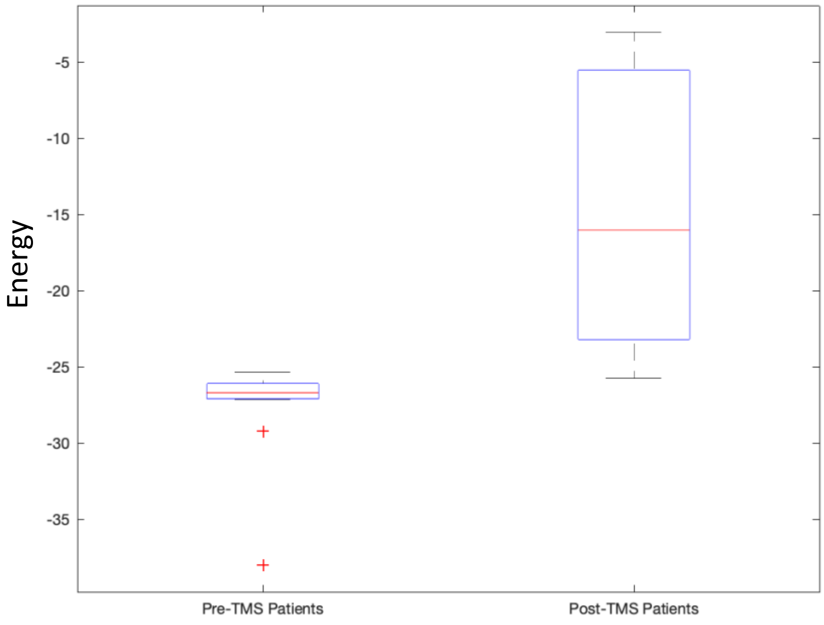

Figure S11 Active salience network state significantly distinguishes energies pre and post TMS subthreshold pulse condition of patients when the vermis is stimulated (p-value = 0.00012)

References

1. Cole, M.W.; Reynolds, J.R.; Power, J.D.; Repovs, G.; Anticevic, A.; Braver, T.S. Multi-Task Connectivity Reveals Flexible Hubs for Adaptive Task Control. Nat Neurosci 2013, 16, 1348–1355, doi:10.1038/nn.3470.
2. Kandel, E.R.; Schwartz, J.H.; Jessell, T.M.; Biochemistry, D. of; Jessell, M.B.T.; Siegelbaum, S.; Hudspeth, A.J. Principles of Neural Science; McGraw-hill New York, 2000; Vol. 4;.
